# Heterogeneity derived from ^18^F‐FDG PET/CT predicts immunotherapy outcome for metastatic triple‐negative breast cancer patients

**DOI:** 10.1002/cam4.4522

**Published:** 2022-03-11

**Authors:** Yizhao Xie, Cheng Liu, Yannan Zhao, Chengcheng Gong, Yi Li, Shihui Hu, Shaoli Song, Xichun Hu, Zhongyi Yang, Biyun Wang

**Affiliations:** ^1^ Department of Medical Oncology Fudan University Shanghai Cancer Center Shanghai China; ^2^ Department of Oncology Shanghai Medical College Fudan University Shanghai China; ^3^ Department of Nuclear Medicine Fudan University Shanghai Cancer Center Shanghai China; ^4^ Center for Biomedical Imaging Fudan University Shanghai China; ^5^ Shanghai Engineering Research Center of Molecular Imaging Probes Shanghai China

**Keywords:** heterogeneity, immunotherapy, PET‐CT, predictors, triple‐negative breast cancer

## Abstract

**Background:**

Recently, immunotherapy has been used to treat metastatic triple‐negative breast cancer (mTNBC). Basic research has indicated a relation between tumor heterogeneity and the immune response. Tumor heterogeneity derived from ^18^F‐FDG PET/CT is a potential predictor of chemotherapy results; however, few studies have focused on immunotherapy. This study aims to develop a convenient and efficient measurement of tumor heterogeneity for the prediction of immunotherapy in mTNBC patients.

**Methods:**

We enrolled mTNBC patients who received immunotherapy (PD‐1/PD‐L1 antibody) plus chemotherapy as first‐line treatment and underwent ^18^F‐FDG PET/CT scans before treatment. We defined a novel index representing tumor heterogeneity calculated from the standard uptake value (SUV) as IATH and IETH. Optimal cutoffs were determined using time‐dependent receiver operator characteristics (ROC) analysis.

**Results:**

A total of 32 patients were enrolled and analyzed in this trial. A significantly longer median PFS was observed in the low SUVmax group than in the high SUVmax group (9.4 vs. 5.8 months, HR = 0.3, 95% CI 0.1–0.9, *p* = 0.025). The median PFS of low‐IATH patients was significantly longer than that of high‐IATH patients (HR = 0.3, 95% CI 0.1–0.8, *p* = 0.022). Similarly, patients with low IETH had significantly longer PFS than patients with high IETH (9.4 vs. 4.9 months, HR = 0.3, 95% CI 0.1–0.7, *p* = 0.01). Multivariate analysis demonstrated IETH as an independent predictor of PFS.

**Conclusions:**

This study proposed a novel method to assess intratumor and intertumor heterogeneity among metastatic breast cancer patients and determined that baseline IETH derived from ^18^F‐FDG PET/CT could represent a simple and promising predictor for first‐line immunotherapy among mTNBC patients.

## Graphical abstract

This study proposed a novel method to assess the intratumor and intertumor heterogeneity among metastatic breast cancer patients and figured out that baseline IETH derived from ^18^F‐FDG PET/CT could be a simple and promising predictor for first‐line immunotherapy among mTNBC patients.

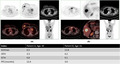

## 1 INTRODUCTION

To date, epidemiological statistics have witnessed an increased incidence of breast cancer compared with lung cancer, and breast cancer has become the most common cancer type worldwide, leading to 2300 new cases and 690 deaths yearly.^1^

Triple‐negative breast cancer (TNBC) is defined as human epidermal growth factor receptor 2 (HER2)‐negative, progesterone receptor (PR)‐negative, and estrogen receptor (ER)‐negative breast cancer, which comprises 15%–20% of all cases and shows a higher recurrence rate as well as worse prognosis than other subtypes with a median survival time of only 1–1.5 years after the diagnosis of metastatic disease.^2^, ^3^, ^4^

Although chemotherapy remains the cornerstone in metastatic TNBC (mTNBC), immunotherapy has recently shown exciting results. A phase III IMpassion130 study enrolled 902 metastatic or inoperable TNBC patients and randomized them into atezolizumab or placebo plus nab‐paclitaxel. The results showed significantly improved progression‐free survival (PFS) in the atezolizumab group.^5^ The KEYNOTE‐355 study compared pembrolizumab or placebo plus chemotherapy in first‐line treatment of mTNBC and indicated prolonged PFS in programmed death‐ligand 1 (PD‐L1)‐positive patients or patients with a combined positive score (CPS) of greater than 10.^6^

Although immunotherapy provides a great opportunity for patients, controversial results in different studies urge us to find more efficient biomarkers and prognostic factors to identify proper candidates for immunotherapy.

Tumor heterogeneity has been demonstrated to predict treatment responses as well as prognosis for malignant tumors.^7^, ^8^ Fluorine‐18 fluorodeoxyglucose positron emission tomography/computed tomography (^18^F‐FDG PET/CT) offers a noninvasive and overall evaluation of tumor heterogeneity and is more convenient and extensive than traditional biopsy methods. Previous studies preliminarily explored the predictive effect of tumor heterogeneity evaluated by PET/CT and determined valuable parameters in the prediction of recurrence, treatment response, and prognosis.^9^, ^10^, ^11^, ^12^, ^13^

Previous studies focused on the prediction of response to chemotherapy and radiotherapy, but immunotherapy has a different mechanism. Moreover, a recent study found that immune cells have a great capacity to take up intratumoral glucose and glutamine; thus, it would be important to identify a relationship between PET/CT and immunotherapy.^9^, ^10^, ^11^, ^12^, ^13^ Thus, our study aims to identify the intertumor and intratumor heterogeneity of mTNBC patients through quantitative parameters derived from PET/CT scans and explore the predictive value of these parameters for first‐line immunotherapy‐based treatment response.

## 2 METHODS

### 2.1 Patients

We retrospectively screened all patients who received immunotherapy (PD‐1/PD‐L1 antibody) plus chemotherapy as first‐line treatment for mTNBC from 2015 to 2021 in Fudan University Shanghai Cancer Center. Patients who underwent whole‐body ^18^F‐FDG PET/CT scans within 30 days before the first regimen were enrolled in this study. Patients with incomplete medical records were excluded.

mTNBC was defined as unresectable, recurrent, or metastatic ER‐, PR‐, and HER2‐breast cancer. ER‐, PR‐, and HER2‐ were defined as ER less than 1%, PR less than 1%, and a score of 0–1+ in HER2 immunohistochemistry (IHC) or IHC 2+ and negative fluorescence in situ hybridization (FISH), respectively. Medical and PET/CT data were collected retrospectively from the electronic medical database system.

The Fudan University Shanghai Cancer Center Institutional Review Boards and Ethics Committee approved this study for clinical investigation. All of the methods were conducted in conformity with the Declaration of Helsinki and relevant guidelines.

### 2.2 PET/CT scan

^18^F‐FDG was generated automatically by a cyclotron. Patients were asked to fast for 6 h or more before the exam, and the blood glucose was less than 10 mmol/L before the injection of ^18^F‐FDG (dose: 3.7 MBq/kg). Patients laid on a comfortable cushion in a quiet room during the injection.

PET/CT scans were acquired on a Siemens mCT Flow PET/CT scanner approximately 60 min after the injection. The coregistered images were displayed and confirmed on a workstation.

### 2.3 Image analysis

The images were evaluated independently by two experienced nuclear medicine physicians with attending certification on a multimodality computer platform. If a discrepancy occurred, a third physician joined the discussion to reach a consensus. Quantification of the tumor glucose metabolic activity was calculated using the standard uptake value (SUV) normalized to body weight. The maximum and mean SUV (SUVmax and SUVmean, respectively) for metastatic lesions were evaluated by manually placing an individual region of interest (ROI) around the lesion on the coregistered and fused transaxial PET/CT images. Lesions less than 8 mm in diameter were not included due to partial volume effects and repeatability. The metabolic tumor volume (MTV) was exported automatically from the manual delineation using software based on an adaptive threshold of SUV intensity >50% of SUVmax within the contouring margin. We propose two novel measures of tumor heterogeneity: intratumor heterogeneity (IATH), which is defined as the largest value of subtraction between SUVmax and SUVmean (SUVmax‐SUVmean) among each lesion, and intertumor heterogeneity (IETH), which is defined as subtraction between SUVmax and SUVmean (SUVmax‐SUVmean) among all lesions.

### 2.4 Statistical analysis

The quantitative data are presented as medians (range) or numbers of patients, and the categorical data are reported as counts (percentage). Descriptive statistics were used to summarize the clinicopathologic characteristics. Treatment outcome was represented by PFS and OS. PFS was measured from treatment initiation to the first confirmed disease progression or death. OS was defined as the time between treatment initiation and death or last follow‐up. The disease‐free interval (DFI) was defined as the time between surgery and diagnosis of metastatic disease. Response Evaluation Criteria in Solid Tumors (RECIST) version 1.1 was used to determine disease progression. The optimal cutoff values of PET/CT parameters and heterogeneity index were determined by time‐dependent survival receiver operating characteristic (ROC) analysis. The high‐value and low‐value groups were discriminated by the optimal cutoff point. Survival rates were estimated using the Kaplan–Meier method and compared using the log‐rank test. Prognostic factors were investigated by a Cox regression model with a 95% confidence interval in both univariate and multivariate models. A *p* value less than 0.05 was considered statistically significant. Statistical analyses were managed using SPSS (IBM) version 23.0 or R language (R i386 4.0.2).

## 3 RESULTS

### 3.1 Patient characteristics

A total of 32 patients met our criteria and were enrolled in our study. All patients were reviewed and evaluated retrospectively. Patients and disease characteristics at baseline are shown in Table 1. The median age of patients was 46 years. Except for three de novo stage IV patients, all patients received surgery and had recurrent disease. All patients were in stable overall condition. A total of 40.6% of patients had more than three metastatic sites and 56.3% of patients had visceral metastases.

**TABLE 1:** Patient characteristics

Characteristics	No. (%)
Median age (range)	46 (31–69)
Menopausal status	
Postmenopausal	15 (46.9)
Premenopausal	17 (53.1)
DFI	
<2 years	17 (53.1)
≥2 years	12 (37.5)
De novo stage IV	3 (9.4)
ECOG score	
0–1	32 (100)
Number of metastatic sites	
1	11(34.4)
2	8 (25.0)
≥3	13 (40.6)
Metastatic sites	
Liver	8 (25)
Lung	12(37.5)
Bone	14(43.8)
Visceral	18(56.3)

### 3.2 Predictive value of baseline characteristics

At the time of analysis, 23 of 32 patients had documented progressive disease and 5 of 32 patients had died. The median PFS was 8.0 months (95% CI 6.1–9.9) and the median OS was not reached.

The predictive value of traditional clinical factors was first analyzed. We determined that the existence of liver metastasis was associated with shorter PFS in univariate analysis (HR = 0.4, 95% CI 0.1–0.9, *p* = 0.03) and a trend of shorter PFS was observed in patients with a DFI less than 2 years (HR = 0.4, 95% CI 0.2–1.1, *p* = 0.08). In multivariate analysis, DFI less than 2 years (*p* = 0.032) was an independent predictor of worse PFS. The detailed evaluation of prognostic factors is shown in Table 2.

**TABLE 2:** Summary of univariate and multivariate PFS analyses

Parameters	No.	Median PFS	Univariate analysis		Multivariate analysis	
		(95% CI)	HR (95% CI)	*p* value	HR (95% CI)	*p* value
Age						
<47	15	7.7 (6.9–8.5)	0.6 (0.3–1.3)	0.21		
≥47	16	7.4 (3.4–11.5)				
DFI						
<2 years	17	5.8 (2.1–9.4)	0.4 (0.2–1.1)	0.08	0.19 (0.04–0.95)	0.039
≥2 years	12	9.4 (7.6–11.3)				
No. of metastatic sites						
1–2	19	8.0 (4.5–11.5)	0.9 (0.4–2.1)	0.81		
≥3	13	6.8 (2.3–11.4)				
Liver metastasis						
Yes	8	3.0 (1.0–6.1)	0.4 (0.1–0.9)	0.03	0.33 (0.09–1.21)	0.094
No	24	8.7 (6.5–10.9)				
Visceral metastasis						
Yes	18	8.7 (2.2–15.3)	0.9 (0.4–2.2)	0.91		
No	14	6.8 (4.7–8.9)				
SUVmax						
≤10.03	16	9.4 (2.6–16.1)	0.3 (0.1–0.9)	0.025	0.91 (0.29–2.83)	0.91
>10.03	16	5.8 (3.0–8.5)				
IATH						
≤3.8	18	9.4 (6.5–12.4)	0.3 (0.1–0.8)	0.022	0.65 (0.06–6.64)	0.71
>3.8	14	5.8 (2.1–9.4)				
IETH						
≤7.5	20	9.4 (7.0–11.8)	0.3 (0.1–0.7)	0.01	0.27 (0.02–0.73)	0.023
>7.5	12	4.9 (2.4–7.4)				
MTV (ml)						
≤2.95	15	9.5 (4.4–14.5)	0.6 (0.3–1.5)	0.3		
>2.95	17	6.8 (3.2–10.4)				

### 3.3 Predictive value of PET parameters

Tumor heterogeneity derived from PET parameters was assessed for predicting PFS. The optimal cutoff values of PET parameters were determined by time‐dependent ROC analysis. The following values were obtained: 10.03 for SUVmax, 2.95 ml for MTV, 3.8 for IATH, and 7.5 for IETH.

**FIGURE 1:**
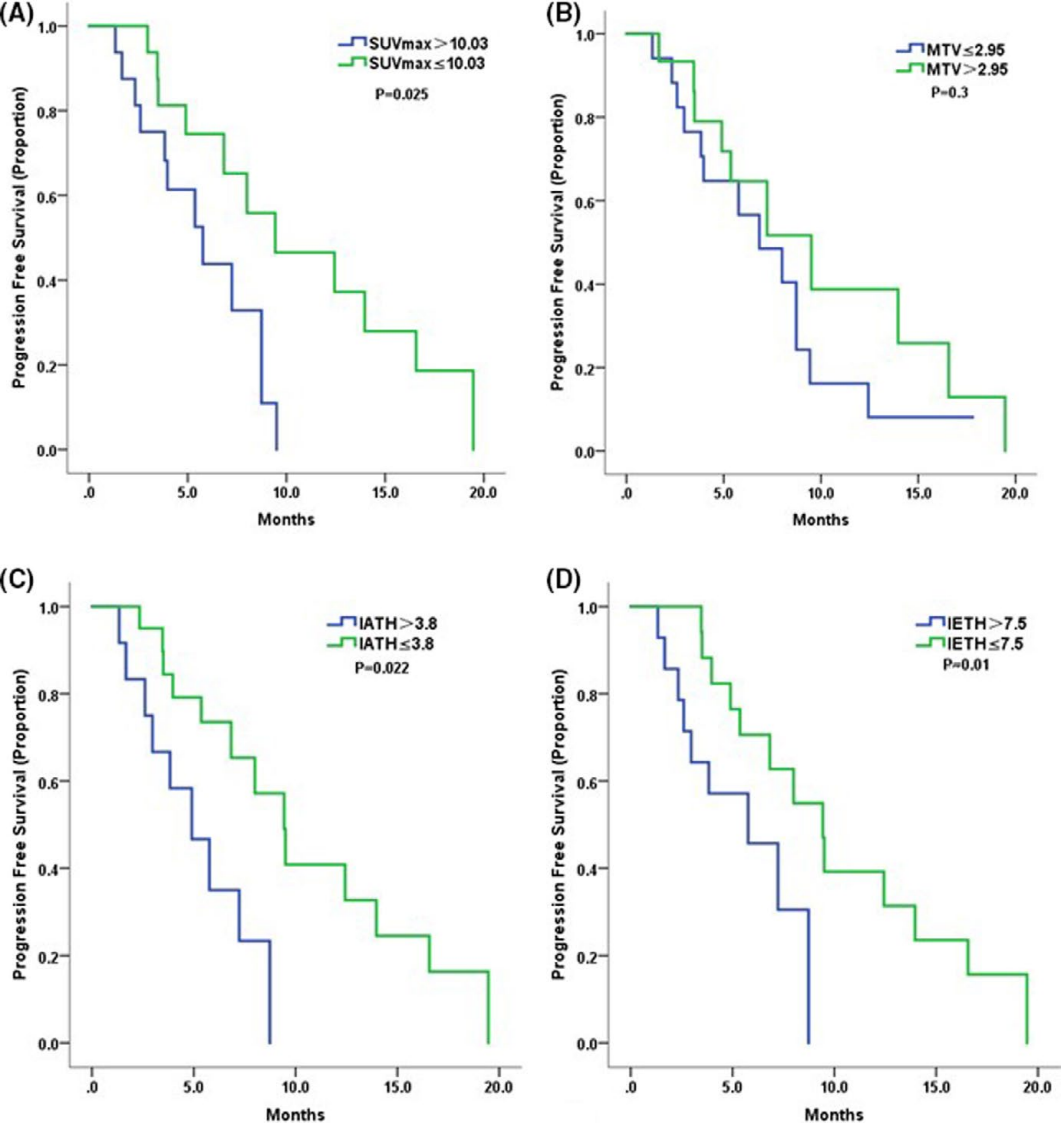
The Kaplan–Meier curves for progression‐free survival based on low and high levels of PET parameters: (A) SUVmax; (B) MTV; (C) IATH; (D) IETH

**FIGURE 2:**
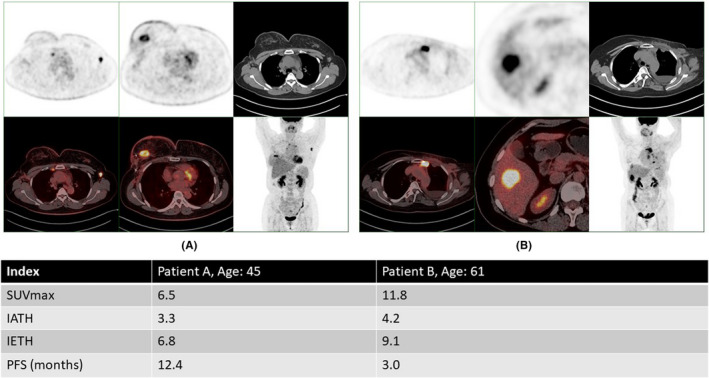
Analysis examples: Patient A was a 45‐year‐old female mTNBC patient. The following heterogeneity parameters of her metastatic lesions were obtained: SUVmax 6.5, IATH 3.3, and IETH 6.8 with a PFS of 12.4 months. Patient B was a 61‐year‐old female mTNBC patient. The following heterogeneity parameters of her metastatic lesions were obtained: SUVmax 11.8, IATH 4.2, and IETH 9.1 with a PFS of 3.0 months

Our results showed that the median PFS of the high SUVmax group was significantly shorter than that of the low SUVmax group (9.4 vs. 5.8 months, HR = 0.3, 95% CI 0.1–0.9, *p* = 0.025, Figure 1A). The median PFS of low‐IATH patients was 9.4 months, which was significantly longer than that of high‐IATH patients (HR = 0.3, 95% CI 0.1–0.8, *p *= 0.022, Figure 1C). Similarly, patients with low IETH had significantly longer PFS than patients with high IETH (9.4 vs. 4.9 months, HR =* *0.3, 95% CI 0.1–0.7, *p *= 0.01, Figure 1D). MTV was not a significant predictor of PFS (Figure 1B). Multivariate analysis identified IETH as an independent predictor of PFS even after balancing the known factors (HR =* *0.27, 95% CI 0.02–0.73, *p *= 0.023). Details of the prognostic are displayed in Table 2. PET analysis of two representative patients is presented in Figure 2. Patient A was a 45‐year‐old female mTNBC patient. The following heterogeneity parameters were obtained for her metastatic lesions: SUVmax 6.5, IATH 3.3, and IETH 6.8 with a PFS of 12.4 months. Patient B was a 61‐year‐old female mTNBC patient. The following heterogeneity parameters were obtained for her metastatic lesions: SUVmax 11.8, IATH 4.2, and IETH 9.1 with a PFS of 3.0 months.

### 3.4 Survival ROC analysis

**FIGURE 3:**
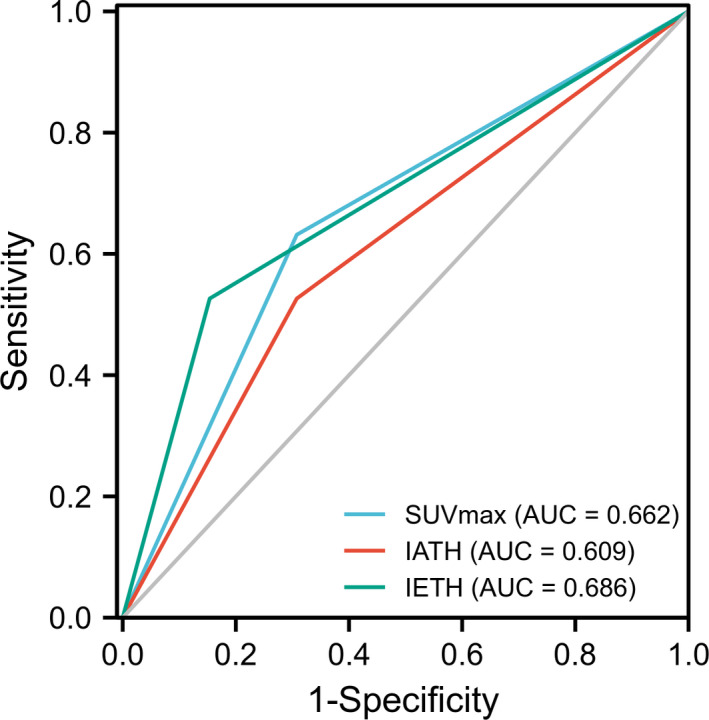
Survival ROC curve

To further evaluate and compare the predictive ability of different parameters, we performed and calculated the time‐dependent ROC curves and area under the curve (AUC) (Figure 3).

Our results showed that SUVmax had an AUC of 0.66, IATH had an AUC of 0.61, and IETH had an AUC of 0.69. Given that the three parameters showed AUCs greater than 0.6, moderate predictive value was confirmed for PFS. Moreover, IETH had an AUC of 0.69, indicating a strong and promising predictive ability in immunotherapy.

## 4 DISCUSSION

This study developed a novel method to assess intratumor and intertumor heterogeneity among metastatic breast cancer patients. We evaluated parameters derived from baseline ^18^F‐FDG PET/CT and determined that higher SUVmax, IATH, and IETH predicted worse PFS in first‐line immunotherapy among mTNBC patients.

Regarding the use of immunotherapy for the treatment of breast cancer, previous studies focused on traditional biomarkers, such as PD‐L1 and tumor‐infiltrating lymphocyte (TIL) status. The Impassion 130 trial used a cutoff of 91% tumor‐infiltrating immune cells with positive PD‐L1 staining and demonstrated a significantly longer OS of the atezolizumab plus nab‐paclitaxel group compared with the control group (25 vs. 18 months, HR = 0.71, 95% CI 0.54–0.94), which was not observed in intention‐to‐treat patients (HR = 0.86, 95% CI 0.72–1.02).^5^ In contrast, the Keynote‐119 study indicated that PD‐L1‐positive patients did not benefit from pembrolizumab monotherapy compared with chemotherapy, suggesting a demand for a higher PD‐L1 cutoff value to select patients.^15^ Furthermore, studies have shown that PD‐1 and PD‐L1 IHC testing can be technically difficult to interpret in terms of different testing methods as well as pathologists, which makes it difficult to select patients precisely.^16^, ^17^ In the Keynote‐119 study, stromal TILs ≥5% could predict benefit from pembrolizumab.^15^ Similarly, CD8+ T‐cell infiltration was demonstrated to predict overall survival benefit with atezolizumab in the IMpassion130 trial.^5^ TILs reflected the predictive potential of immunotherapy, but this marker is not widely and standardly used in hospitals.

Our study provided a relatively objective method that was calculated from PET/CT images as a predictor of immunotherapy. This method could avoid inconformity among different testing methods or different physicians.

Tumor heterogeneity correlates with the cancer microenvironment, immune infiltration, cancer metastasis, and drug resistance.^18^ Heterogeneity data derived from ^18^F‐FDG PET/CT were explored in the prediction of recurrence, prognosis, and chemotherapy and radiotherapy outcomes among different cancer types.^9^, ^12^, ^19^, ^20^, ^21^ However, most research emphasized intratumor heterogeneity, whereas our study raised a novel concept of calculating intertumor heterogeneity. This parameter could evaluate the overall heterogeneity of metastatic disease. Moreover, due to the limited use of immunotherapy in breast cancer, this is the first study aiming to identify an association between immunotherapy and the heterogeneity index among breast cancer patients.

This study determined the predictive ability of SUVmax, IATH, and IETH in the context of first‐line immunotherapy in mTNBC patients. The concept of IATH and IETH was first raised in this study. We further demonstrated IETH as an independent predictor after balancing other factors. In the survival ROC analysis, IETH also showed the highest AUC of 0.69 compared to the other parameters. In contrast, MTV did not show predictive power, thereby excluding the confounding factor of tumor size. Our findings could provide clinical doctors with a convenient method to identify potential patients sensitive to immunotherapy‐based treatment.

Recent studies have explored the mechanism by which tumor heterogeneity influences immunotherapy. A study found that higher intratumor heterogeneity in melanoma led to an inhibited immunotherapy response likely due to of the loss of immunogenicity and reduced T‐cell infiltration.^22^ Another study using an online database suggested that higher heterogeneity calculated using a mutant‐allele tumor heterogeneity algorithm correlated with less immune response and worse survival in a breast cancer cohort.^23^ A recent interesting study indicated that myeloid cells and T cells exhibited a greater capacity to take up intratumoral glucose than cancer cells. This finding was consistent with the results of our study that indicated that higher glucose uptake heterogeneity means a greater imbalance in T‐cell contribution, leading to failure of immunotherapy.^14^ Given that tumor heterogeneity exhibits a strong relationship with immunotherapy, more efforts should be made to reverse heterogeneity.

The limitations of the present study should be noted. On the one hand, this study enrolled a small cohort of Asian patients, and further prospective trials with a large cohort are warranted to confirm our results. On the other hand, tumor heterogeneity involves more complex mechanisms in addition to glucose metabolism that might not be completely revealed by PET/CT. More translational and clinical research is needed to uncover the best method for evaluating tumor heterogeneity.

## 5 CONCLUSION

This study proposed a novel method to assess the intratumor and intertumor heterogeneity among metastatic breast cancer patients and determined that baseline IETH derived from ^18^F‐FDG PET/CT could be a simple and promising predictor for first‐line immunotherapy among mTNBC patients.

## CONFLICT OF INTEREST

The authors declare that they have no conflict of interest.

## AUTHOR CONTRIBUTIONS

Yizhao Xie collected all of the data, performed statistical analysis, and completed the manuscript. Cheng Liu, Zhongyi Yang, and Shaoli Song analyzed and confirmed the PET figures. Chengcheng Gong, Yi Li, Yannan Zhao, and Shihui Hu participated in the data collection. Xichun Hu, Biyun Wang, and Zhongyi Yang designed and performed the study and revised the manuscript.

## ETHICS APPROVAL

All of the methods were conducted in conformity with the Declaration of Helsinki and relevant guidelines. The study was exempted from written informed consent and required ethics approval from the Institutional Review Board of Fudan University Cancer Hospital because it was a retrospective study.

## Data Availability

The datasets generated and/or analyzed during the current study are not publicly available due to hospital policy but are available from the corresponding author upon reasonable request.
